# Modelling climate impacts on paediatric sepsis incidence and severity in Bangladesh

**DOI:** 10.7189/jogh.14.04107

**Published:** 2024-07-19

**Authors:** Gazi MS Mamun, Katelyn Moretti, Farzana Afroze, Ben J Brintz, Abu SMMH Rahman, Monique Gainey, Monira Sarmin, Shamsun N Shaima, Mohammod J Chisti, Adam C Levine, Stephanie C Garbern

**Affiliations:** 1 Infectious Diseases Division, International Centre for Diarrhoeal Disease Research, Bangladesh, Dhaka, Bangladesh; 2 Department of Emergency Medicine, Warren Alpert Medical School of Brown University, Providence, Rhode Island, USA; 3 Nutrition Research Division, International Centre for Diarrhoeal Disease Research, Bangladesh, Dhaka, Bangladesh; 4 Division of Epidemiology, Internal Medicine, University of Utah, Salt Lake City, Utah, USA; 5 Rhode Island Hospital, Providence, Rhode Island, USA

## Abstract

**Background:**

Sepsis is a leading cause of paediatric mortality worldwide, disproportionately affecting children in low- and middle-income countries. The impacts of climate change on the burden and outcomes of sepsis in low- and middle-income countries, particularly in paediatric populations, remain poorly understood. We aimed to assess the associations between climate variables (temperature and precipitation) and paediatric sepsis incidence and mortality in Bangladesh, one of the countries most affected by climate change.

**Methods:**

We conducted retrospective analyses of patient-level data from the International Centre for Diarrhoeal Disease Research, Bangladesh, and environmental data from the National Oceanic and Atmospheric Administration. Using random forests, we assessed associations between sepsis incidence and sepsis mortality with temperature and precipitation between 2009–22.

**Results:**

A nonlinear relationship between temperature and sepsis incidence and mortality was identified. The lowest incidence occurred at an optimum temperature of 26.6°C with a gradual increase below and a sharp rise above this temperature. Higher precipitation levels showed a general trend of increased sepsis incidence. A similar distribution for sepsis mortality was identified with an optimum temperature of 28°C.

**Conclusions:**

Findings suggest that environmental temperature and precipitation play a role in paediatric sepsis incidence and sepsis mortality in Bangladesh. As children are particularly vulnerable to climate impacts, it is important to consider climate change in health care planning and resource allocation, especially in resource-limited settings, to allow for surge capacity planning during warmer and wetter seasons. Further prospective research from more globally representative data sets will provide more robust evidence on the nature of the relationships between climate variables and paediatric sepsis worldwide.

Sepsis is the leading cause of child mortality worldwide and is responsible for over 60% of all paediatric deaths [1]. It is defined as life-threatening organ dysfunction caused by a dysregulated host response to infection. It encompasses a continuum that ranges from sepsis to severe sepsis, septic shock, multiple organ dysfunction syndrome and death [1–7]. Low- and middle-income countries bear a disproportionately high burden of paediatric sepsis, accounting for over 80% of global sepsis-related deaths from infectious diseases such as pneumonia, diarrhoea, and malaria [2–8]. While paediatric sepsis mortality has been decreasing over time, the reduction in sepsis mortality in low- and middle-income countries has been slower compared to high-income countries [4,6,9–12].

The 2022 Intergovernmental Panel on Climate Change reported that without rapid action to address climate change, increases in infectious disease incidence and mortality rates are imminent [8]. Climatic change is linked to infectious disease transmission through various mechanistic pathways, including rising temperatures, increased flooding and droughts, and changing habitats. Such impacts can trigger outbreaks, such as enteric infections leading to diarrhoea (e.g. cholera, rotavirus), respiratory diseases (e.g. pneumonia, influenza), and vector-borne diseases (e.g. malaria, dengue) [11]. Recent studies from India have found that the daily mean temperature was strongly associated with child mortality [13] and childhood stunting [14]. Climate is an important driver of spatial and seasonal patterns of infections, year-to-year variations in incidence (including epidemics), and longer-term shifts in at-risk populations [8]. Given the anticipated increases in infectious disease incidence and mortality with climate change, there is an urgent need to understand how climate and weather variations impact the burden of sepsis among paediatric populations globally. While rises in certain infectious diseases such as respiratory and enteric infections have been linked to rising temperatures and humidity, there is a paucity of literature on the effects of climate change on paediatric sepsis and sepsis outcomes in low-resource settings, despite both the greater vulnerability to climate change and higher rates of child mortality from sepsis in these settings [15].

Bangladesh, one of the most vulnerable countries to climate change impacts [16], has experienced increased extreme weather and a sustained rise in temperature and precipitation. These shifts have increased the prevalence and epidemiology of infectious diseases, particularly enteric pathogens causing regular seasonal diarrheal outbreaks and dengue and malaria [12,15,17]. Prior research from the International Centre for Diarrhoeal Disease Research, Bangladesh (icddr,b) found climate variability strongly linked with childhood diarrhoea and pneumonia incidence. The influence of environmental factors, such as temperature and rainfall, varies by season and location and exerts a more pronounced effect in patients with lower socioeconomic status and limited access to sanitation and hygiene [15,18].

In this study, the impacts of environmental temperature and precipitation on paediatric sepsis incidence and mortality were assessed for children admitted to the icddr,b Dhaka Hospital between 2009–22.

## METHODS

### Study design, setting, and population

We performed cross-sectional analyses correlating environmental temperature and precipitation to sepsis incidence and mortality. We collected deidentified data from icddr,b’s electronic health record system on sepsis incidence among all paediatric patients under 18 years of age admitted to the icddr,b Dhaka Hospital from 2009 to 2022. Dhaka Hospital is a specialised non-profit hospital that provides care to nearly 200 000 patients annually with diarrhoea and other infectious diseases [19]. Dhaka Hospital serves a primarily impoverished population and treats a high volume of paediatric patients with sepsis, with children younger than five years making up nearly 60% of all patients [19]. Patients with sepsis are admitted to either the intensive care unit or general hospital ward, depending on the severity of their illness. The vast majority of paediatric patients admitted with sepsis to iccdr,b have enteric (in particular cholera and rotavirus) or respiratory infections as the underlying source of sepsis; these infections are highly susceptible to climate impacts such as rises in temperature, rainfall, and humidity [17].

### Ethical approval

Ethical approval for this study was obtained from Rhode Island Hospital’s Institutional Review Board (1760162) and icddr,b’s Research Review Committee and Ethical Review Committee (PR-21062).

### Data collection

#### Patient data

All data required for patient care at icddr,b are routinely entered by clinicians into the SHEBA electronic medical record (SHEBA EMR), including sociodemographic, laboratory, and clinical data. We extracted the following variables for all patients that had <18 years: final diagnosis, final mortality outcome, initial vital signs and laboratory parameters (when available), gender, age, and nutrition status (calculated using a weight-for-age Z score (WHZ) less than –2 as a measure of acute malnutrition and a height-for-age Z score (HAZ) less than –2 as a measure of chronic malnutrition).

Sepsis incidence was determined through identification of patients who met the Goldstein definition of paediatric sepsis, as defined by an infectious diagnosis and at least two of the following four criteria [18], one of which must be abnormal temperature or leukocyte count: core temperature of >38.5°C or <36°C; heart rate >2 standard deviations above the mean, or for children <1 year a mean heart rate <10th percentile for age; respiratory rate >2 standard deviations above the mean, mechanical ventilation in the setting of respiratory distress; and abnormal leukocyte count for age.

We used leukocyte abnormalities resulting on the same day as vital sign abnormalities collected from the SHEBA EMR to determine if sepsis criteria were met. As a sensitivity analysis, models were also fit for patients explicitly diagnosed with sepsis by a physician in the SHEBA EMR. At icddr,b, physicians diagnose sepsis only after the correction of dehydration and tend to be more selective in whom they diagnose with sepsis [20], compared to the more sensitive but less specific Goldstein definition. Sepsis mortality was defined as a patient who had a date of death recorded.

#### Weather data

Weather data was obtained from the National Oceanic and Atmospheric Administration reporting weather stations 419 230 and 419 220. These two Dhaka stations reported the most complete temperature and precipitation data (1.74 km and 7.43 km from icddr,b Dhaka Hospital). We combined the station data by averaging daily temperature and precipitation values across the two stations, excluding missing values. Finally, we calculated an average across a moving window of 14 days of lagged exposure for each weather variable, excluding missing values. Given the temperature range in Dhaka, the majority of health impacts were thought to be heat-related. Therefore, we selected a conservative lag of 14 days to capture heat impacts as well as the majority of any cold impacts [21,22]. This resulted in only three missing precipitation values and no missing temperature values between 2009–22 (just 0.014% of all weather data for this period). We used the moving averages for temperature and precipitation as independent variables in the sepsis incidence and mortality analyses.

#### Analysis

We conducted univariable analyses for each covariate of interest for sepsis incidence and mortality outcomes using the Wilcoxon rank sum test or χ^2^ test, as appropriate. Covariates included averaged windows of lagged exposure to temperature and precipitation and patient-specific covariates (gender, age, WHZ, HAZ). Given the nonlinear, non-monotonic relationship between temperature and sepsis, a random forest model provided further insight into the interactions between these covariates of interest and sepsis incidence and mortality. Unlike regression modelling, which presupposes a specific type of relationship between variables (i.e. linear, logistic, quadratic, etc.) and the presence of interactions between variables, random forests allow us to identify the potentially complex relationships between climate and sepsis variables without necessarily knowing in advance the nature of those relationships. Specifically, we produced partial dependency plots to visualise the marginal effect of each covariate on the probability of sepsis incidence or sepsis mortality. We utilised bootstrapping to produce 95% confidence intervals for the marginal probability of sepsis dependent on temperature and precipitation. We conducted a sensitivity analysis by repeating all analyses using the alternate definition of sepsis (i.e. patients diagnosed by a physician with sepsis in the SHEBA EMR).

## RESULTS

A total of 71 314 records were included in the data set, of which 50 629 had sufficient data to calculate a WHZ, and 51 335 had sufficient data to calculate a HAZ (**Table 1**). Among children admitted to the icddr,b Dhaka Hospital between 2009–22, the average age was 294 days (9.7 months), and 38% were female. There were 18 540 (26%) records satisfying the primary definition of sepsis (using the Goldstein criteria) and 3067 (4.3%) receiving the clinical diagnosis of sepsis as documented by a physician in the SHEBA EMR.

**Table 1:** Demographics and clinical characteristics of patients in the SHEBA data set*

Characteristics	Patients (n = 71 314)
Age in days, MD (IQR)	294 (167, 519)
Gender	
*Female*	27 411 (38)
*Male*	43 903 (62)
Sepsis	18 540 (26)
*Mortality*	785 (4.2)
Clinically diagnosed sepsis	3067 (4.3)
*Mortality*	830 (27.1)
WHZ, MD (IQR)	–2.01 (–3.10, –0.86)
*Unknown*	20 685 (29)
HAZ, MD (IQR)	–2.14 (–3.43, –0.95)
*Unknown*	19 979 (28)

HAZ – height-for-age Z score, IQR – interquartile range, MD – median, WHZ – weight-for-age Z score

*Presented as n (%) unless specified otherwise.

### Associations with sepsis incidence

**Figure 1:**
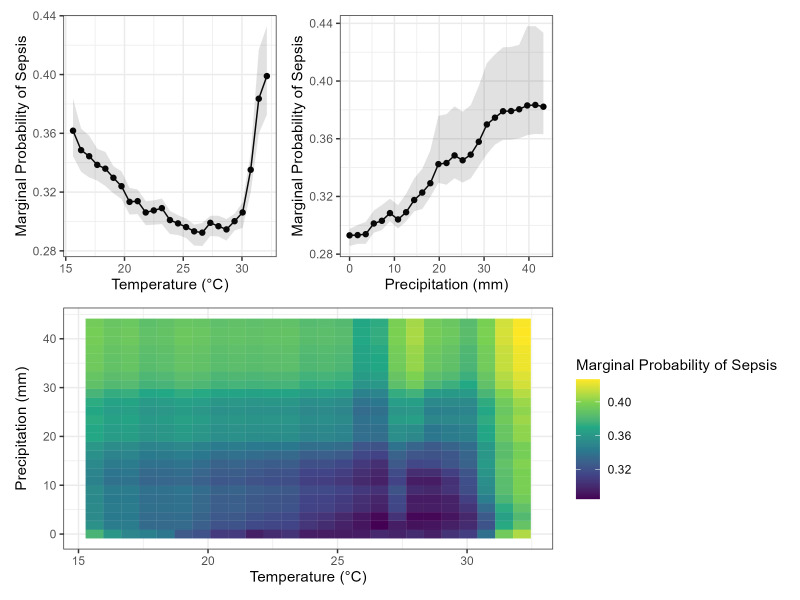
Partial dependency plots showing the marginal probability of sepsis for temperature and precipitation (top) and their interaction (bottom).

In univariable analysis, higher average temperature and precipitation levels were associated with higher sepsis incidence (*P* < 0.001) (**Table 2**). Undernutrition, as calculated by either a lower WHZ or HAZ less than –2, was also associated with higher sepsis incidence (*P* < 0.001). Using partial dependency plots to visualise the marginal probabilities of sepsis incidence from each covariate in the random forest model, a nonlinear relationship between sepsis, temperature and precipitation was identified with a sharp rise in sepsis incidence above an optimum temperature of 26.6°C where sepsis incidence reached a nadir and a more gradual rise in sepsis incidence as temperatures cooled from the optimum. In addition, an interaction between temperature and precipitation was noted, with the highest sepsis incidence occurring during periods of high temperature and high precipitation (**Figure 1**).

**Table 2:** Univariable comparison of those with and without sepsis based on the Goldstein definition*

Characteristics	No sepsis (n = 52 774)	Sepsis (n = 18 540)	*P*-value
Age in days	299 (168, 518)	286 (165, 547)	0.8
Gender, n (%)			>0.9
*Female*	20 284 (38)	7127 (38)	
*Male*	32 490 (62)	11 413 (62)	
Temperature in °C	27.9 (23.3, 29.5)	28.3 (23.8, 29.5)	<0.001
Precipitation in mm	0.2 (0.0, 4.5)	0.8 (0.0, 5.7)	<0.001
WHZ	–1.93 (–3.03, –0.77)	–2.24 (–3.30, –1.11)	<0.001
HAZ	–2.11 (–3.36, –0.94)	–2.23 (–3.61, –0.98)	<0.001

HAZ – height-for-age Z score, WHZ – weight-for-age Z score

*Presented as MD (IQR) unless specified otherwise.

### Sensitivity analysis for sepsis incidence

In the univariable analysis using the alternative (physician-diagnosed) definition of sepsis, there was evidence of an association between sepsis and each of the covariates considered (**Table 3**). Sepsis incidence was greatest for higher environmental temperatures. In addition, those with sepsis were younger and had a lower WHZ and a lower HAZ. Contrary to the primary model, precipitation was negatively correlated with sepsis in the univariable analysis, with less precipitation associated with higher sepsis incidence. Using partial dependency plots to visualise the marginal probabilities of sepsis incidence in the random forest model, a nonlinear relationship between sepsis, temperature and precipitation was again identified. In addition, an interaction between temperature and precipitation was noted, with the highest sepsis incidence occurring during periods of high temperature and high precipitation (Figure S1 in the **Online Supplementary Document**).

**Table 3:** Univariable comparison of those with and without clinically diagnosed sepsis*

Characteristics	No sepsis (n = 68 247)	Sepsis (n = 3 067)	*P*-value
Age in days	303 (170, 525)	214 (101, 410)	<0.001
Gender, n (%)			<0.001
*Female*	26 068 (38)	1343 (44)	
*Male*	42 179 (62)	1724 (56)	
Temperature in °C	28.0 (23.4, 29.5)	28.4 (23.7, 29.6)	<0.001
Precipitation in mm	0.3 (0.0, 4.8)	0.1 (0.0, 4.3)	<0.001
WHZ	–2.00 (–3.10, –0.85)	–2.13 (–3.34, –1.04)	<0.001
HAZ	–2.13 (–3.42, –0.94)	–2.38 (–3.86, –1.21)	<0.001

HAZ – height-for-age Z score, WHZ – weight-for-age Z score

*Presented as MD (IQR) unless specified otherwise.

### Associations with sepsis mortality

**Figure 2:**
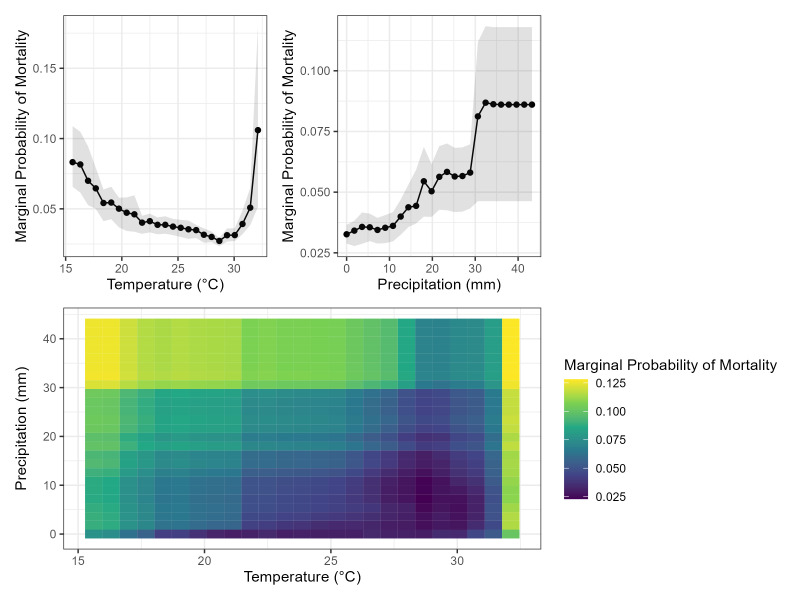
Partial dependency plots showing the marginal probability of mortality in the sepsis population for temperature and precipitation (top) and their interaction (bottom).

In univariable analysis, those who died of sepsis were younger and had lower WHZ and HAZ scores (**Table 4**). Using partial dependency plots to visualise the marginal probabilities of mortality, a sharp rise in mortality was seen above an optimum temperature of 28°C. Additionally, a slower increase in mortality was observed as precipitation increased. Interaction between temperature and precipitation was again observed, with the highest marginal probability of mortality occurring during both lower temperature and higher precipitation periods and higher temperature and higher precipitation periods (**Figure 2**).

**Table 4:** Univariable comparison of those with and without sepsis mortality based on the Goldstein definition of sepsis*

Characteristics	No sepsis mortality (n = 17 756)	Sepsis mortality (n = 784)	*P*-value
Age in days	291 (167, 548)	214 (120, 365)	<0.001
Gender, n (%)			0.002
*Female*	6785 (38)	342 (44)	
*Male*	10 971 (62)	442 (56)	
Temperature in °C	28.3 (23.8, 29.5)	28.1 (23.2, 29.5)	0.3
Precipitation in mm	0.8 (0.0, 5.7)	0.2 (0.0, 4.3)	<0.001
WHZ	–2.23 (–3.28, –1.10)	–2.90 (–3.98, –1.39)	<0.001
HAZ	–2.21 (–3.57, –0.97)	–3.26 (–4.43, –1.94)	<0.001

HAZ – height-for-age Z score, WHZ – weight-for-age Z score

*Presented as MD (IQR) unless specified otherwise.

In the univariable analysis using the alternative (physician-diagnosed) definition of sepsis, the association with lower WHZ and HAZ scores was preserved. However, there was no difference in age (**Table 5**). Using partial dependency plots to visualise the marginal probabilities of mortality, a sharp rise in mortality was again seen above an optimum temperature of 28°C. Additionally, a slower increase in mortality was observed above an optimum precipitation of seven millimetres per day. An interaction between temperature and precipitation was again observed, with the highest marginal probability of mortality occurring during both lower temperature and higher precipitation periods and higher temperature and higher precipitation periods (Figure S2 and Tables S1–2 in the **Online Supplementary Document**).

**Table 5:** Univariable comparison of those with and without sepsis mortality based on a clinical diagnosis of sepsis*

Characteristic	No sepsis mortality (n = 2237)	Sepsis mortality (n = 830)	*P*-value
Age in days	215 (97, 458)	212 (107, 359)	0.10
Gender, n (%)			0.4
*Female*	970 (43)	373 (45)	
*Male*	1267 (57)	457 (55)	
Temperature in °C	28.4 (23.9, 29.6)	28.3 (23.2, 29.6)	0.15
Precipitation in mm	0.1 (0.0, 4.4)	0.1 (0.0, 4.0)	0.8
WHZ	–2.03 (–3.14, –1.00)	–2.77 (–3.87, –1.27)	<0.001
HAZ	–2.18 (–3.59, –1.05)	–3.28 (–4.51, –1.93)	<0.001

HAZ – height-for-age Z score, WHZ – weight-for-age Z score

*Presented as MD (IQR) unless specified otherwise.

## DISCUSSION

Infectious diseases, such as acute respiratory illnesses and diarrheal diseases, have been previously linked to environmental factors such as temperature and precipitation [16,23,24]. Another recent study from India stated that climate change produced significant health impacts manifested in the rise of both communicable and non-communicable diseases [25]. This retrospective data analysis found the first evidence of associations between temperature and precipitation and the most severe outcomes of paediatric infectious diseases in children, including sepsis and mortality, in children admitted to the icddr,b Dhaka Hospital in Dhaka, Bangladesh over 13 years between 2009–22.

First, a nonlinear (V-shaped) association between temperature and sepsis incidence was identified, in which the lowest sepsis incidence was observed at an optimum temperature of 26.6°C. As temperatures cooled from this optimum temperature, there was a gradual rise in sepsis incidence with a 6.9% absolute change in the marginal risk difference between 26.6–15.6°C. As temperatures warmed, there was a sharp rise in sepsis incidence with a 9.3% absolute change in the marginal risk difference between 30.1–32.1°C. A similar association was found when modelling sepsis mortality in children with sepsis based on either the Goldstein criteria or a clinical definition of sepsis. Similar distributions have been demonstrated previously in temperature-mortality studies, adding external validity to these results [26].

Sepsis incidence was significantly correlated with precipitation in univariable comparisons. In addition, the highest marginal probabilities of sepsis and sepsis mortality occurred when the extreme ends of the temperature range (15.6°C and 32.1°C) coincided with the highest precipitation levels. This finding suggests that the combination of extreme temperature, on either end of the spectrum, and substantial precipitation may contribute to an increased risk of sepsis and sepsis mortality in the paediatric population of Bangladesh. In the sensitivity analysis utilising clinically diagnosed sepsis, the association between precipitation and sepsis incidence was reversed in the univariable analysis. However, the overall trends were similar in our multivariable random forest model. We presume the relationship between sepsis incidence and precipitation is not linear with significant interaction with temperature, limiting the utility of univariable analysis or linear models.

Children represent a particularly vulnerable population to climate impacts [27]. Specifically, in Southeast Asia, undernutrition, acute respiratory infection, diarrheal diseases, low birth weight, and premature mortality have been previously attributed to climate change [28]. As temperatures rise and extreme precipitation events become more common, an additional impact to consider will be a rise in the incidence of paediatric sepsis. In 2020, Dhaka’s population was 21 million, a quarter of under 14 years. Dhaka’s population is expected to reach 31 million by 2035 [29]. The Intergovernmental Panel on Climate Change identified heat, urban heat islands, and extreme rain as key risks to Dhaka as climate change worsens [28].

Past research has found increasing paediatric morbidity from diarrheal diseases with rising environmental temperatures, although data remain limited, and temperature thresholds and the exposure-response function remain undefined [24]. Previous research on the paediatric population of Bangladesh specifically found an association between higher temperatures, the heat humidity index, and the incidence of illness necessitating hospital referral, consistent with the results of this study [30]. Conversely, a different study correlated rising neonatal mortality in Bangladesh with cooler temperatures, finding neonatal mortality was lower in warmer months. While authors concluded that neonates may be more susceptible to hypothermia as opposed to hyperthermia, this study used data from 1982 to 2000. Up-to-date research is greatly needed using more recent data, given Bangladesh’s significant increases in heat emergencies since that time. In April 2023, Bangladesh experienced its hottest temperature in decades, measured at 40.6°C [31]. This occurred in the setting of severe heat waves that swept Southeast Asia. As demands for electricity increased, health consequences were exacerbated by national power outages, limiting adaptive strategies such as air conditioning [32]. Thus, changing extremes may have altered the relative risk of hyper and hypothermia in neonates based on the data collected in the earlier study.

Those living in the world’s poorest communities, particularly children and the elderly, are likely to suffer the most from increases in climate-susceptible infectious diseases [8,12]. Climate change is often described as a threat magnifier, worsening underlying conditions and vulnerabilities through various cascading impacts. Results from this study support the evidence base for the impact of climate and changing climate trends on critically ill children in Bangladesh. Results are likely applicable to other similar low- and middle-income countries in Southeast Asia. This research may help inform future clinical prediction models for sepsis severity and risk of death, thereby helping clinicians better adapt their practice to the care of patients under different weather conditions.

According to the Intergovernmental Panel on Climate Change predictions, days above 35°C in Bangladesh will increase by a median of 11 days by 2040 and 16 days by 2060, even with our most aggressive mitigation strategies, such as reaching net zero emissions globally by 2050. If we consider a future where mitigation is less successful, such as the shared socioeconomic pathways 3–7.0, a projection within the Intergovernmental Panel on Climate Change Sixth Assessment Report that assumes high greenhouse gas emissions in the future (doubling of CO_2_ emissions by the year 2100) [33], the number of days above 35°C increases by 19 days by 2060. Given the risk difference for warmer temperatures found in this study, we predict a significant rise in paediatric sepsis incidence and mortality in Bangladesh as the effects of climate change worsen [34]. More research on the intersection of climate and sepsis, particularly in children, may allow researchers, clinicians, and policymakers to best plan how to strengthen health care systems, better allocate scarce human and material resources, and develop interventions to support patients and communities in adapting and mitigating these public health threats.

At icddr,b Dhaka Hospital, the clinical diagnosis of sepsis was made only in the absence of dehydration or after dehydration was resolved. In 2010, a new sepsis protocol was introduced at icddr,b to help clinicians better recognise sepsis in children, which had been underdiagnosed previously. Thus, it is likely that both physician’s awareness and documentation of sepsis increased over the time period of this study. In addition, some clinicians may have diagnosed sepsis in a patient but failed to record it in the SHEBA EMR. However, this should not have affected our primary definition of sepsis based on the more objective Goldstein criteria. Overall, the incidence of sepsis in our population was much higher using the Goldstein criteria (26%) than the documented physician diagnosis of sepsis (4.3%), suggesting that the Goldstein criteria were likely more sensitive. In contrast, the recorded physician diagnosis represented a more specific (and more severely ill) population of children with sepsis. While the definition of paediatric sepsis continues to evolve, we utilised the Goldstein criteria for this analysis as it represents the internationally accepted definition of paediatric sepsis at the time of this study.

One-quarter of the patients were missing the data necessary to calculate the WHZ and HAZ scores. Lower scores, indicative of malnutrition, were associated with higher sepsis incidence as well as higher sepsis mortality. However, no trends in the missingness of WHZ or HAZ scores throughout the study were observed, suggesting that the data were missing at random and not directly associated with our climate variables or patient outcomes. Also, data were included from a single non-profit referral hospital and may not represent all children admitted to hospitals across Bangladesh during this period. While the Dhaka Hospital provides care for children with all infectious diseases, it is known for its high-quality diarrheal care. Therefore, the aetiology of sepsis may be skewed towards children with diarrheal aetiology.

## CONCLUSIONS

Higher environmental temperatures and higher precipitation two weeks before illness were associated with higher sepsis incidence and mortality in children admitted to the icddr,b Dhaka Hospital in Bangladesh. In addition, important interactions were identified between temperature and precipitation, such that the combination of high temperature and high precipitation, as predicted by current climate models, will likely produce the worst outcomes for children with infections in Bangladesh. Further multi-country research on the mechanisms through which climate variables, infectious diseases, and sepsis interact is needed, especially in paediatric populations from a variety of different settings globally. This improved understanding holds the potential to enable researchers, clinicians, and policymakers to strategically adapt health care systems and mitigate the potential increases in the global burden of paediatric sepsis and mortality due to climate change. This involves optimising resource allocation, both in terms of manpower and materials, and devising interventions to aid patients and communities in adapting to and mitigating these looming public health threats [8]. Presently, climate change is expected to disproportionately impact our most vulnerable populations globally, yet its effects on the health of these vulnerable populations remain insufficiently studied and acknowledged.

## Additional material


Online Supplementary Document
