# Supplementary material for: Modelling climate impacts on paediatric sepsis incidence and severity in Bangladesh
Source: J Glob Health. 2024 Jul 19;14:04107. doi: 10.7189/jogh.14.04107 (PMC11257703; doi:10.7189/jogh.14.04107)
Supplement: Online Supplementary Document [file jogh-14-04107-s001.pdf]

Online Supplementary Documents (OSD)

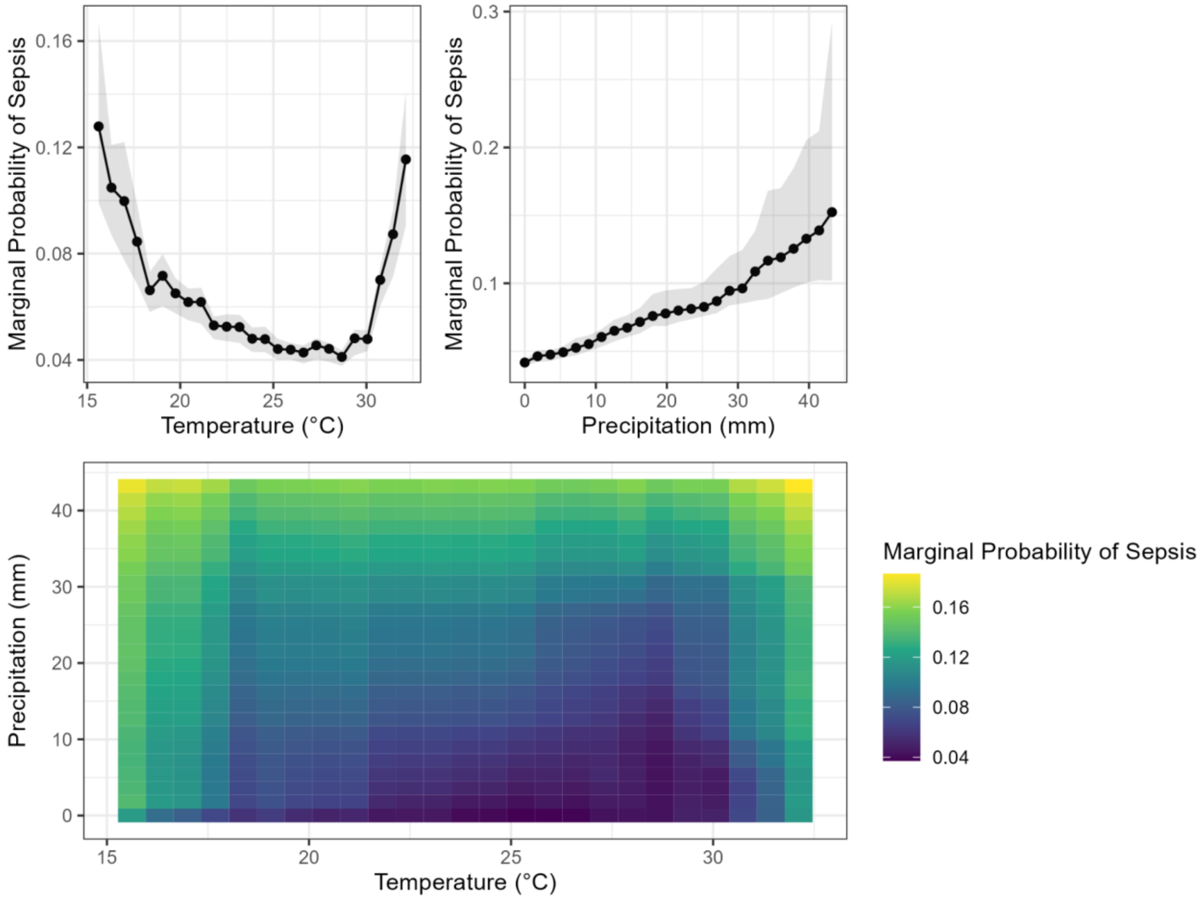

Supplemental Figure S1: Partial Dependency Plots showing the marginal probability of sepsis based on clinician diagnosis for temperature and precipitation (top), and their interaction (bottom).

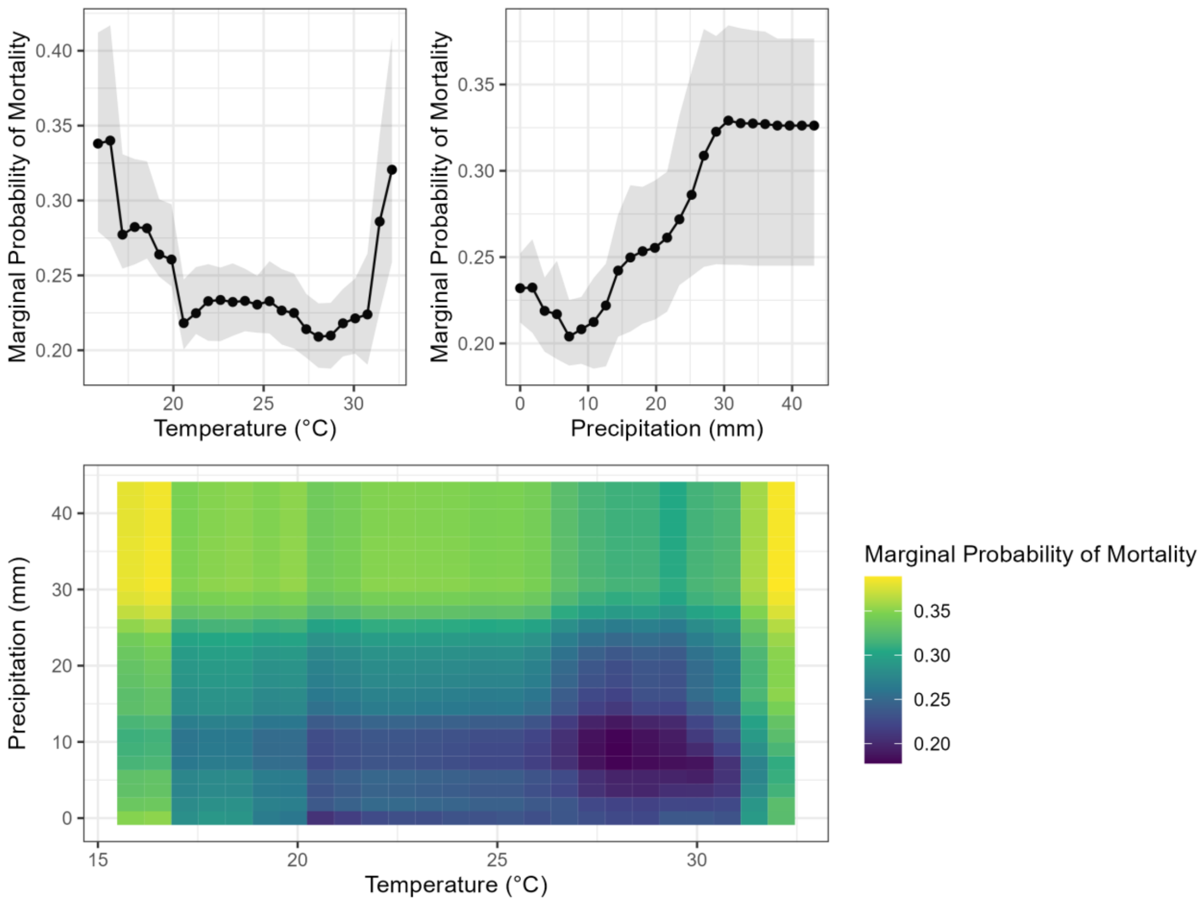

Supplemental Figure S2: Partial Dependency Plots showing the marginal probability of mortality in the sepsis criteria #2 population for temperature and precipitation (top), and their interaction (bottom)

Supplemental Table S1: Results from logistic regression using clinically diagnosed sepsis using both an unadjusted simplified model and patient-specific adjusted model

	Sepsis 2: Simplified			Sepsis 2: Adjusted		
**Characteristic**	**OR**	**95% CI**	**p-value**	**OR**	**95% CI**	**p-value**
Date (scaled)	1.32	1.27, 1.37	<0.001	1.34	1.27, 1.40	<0.001
Temperature	1.02	1.01, 1.04	<0.001	1.02	1.01, 1.03	0.007
Precipitation	0.90	0.74, 1.09	0.3	0.87	0.68, 1.10	0.3
Temperature x Precipitation	1.00	1.00, 1.01	0.3	1.00	1.00, 1.01	0.3
Age (days)				1.00	1.00, 1.00	<0.001
WHZ				0.89	0.86, 0.92	<0.001
HAZ				0.93	0.90, 0.95	<0.001

OR = Odds Ratio, CI = Confidence Interval

Supplemental Table S2: Results from logistic regression using Mortality with the Sepsis Criteria 2 population for both the patient-specific adjusted model and an unadjusted simplified model

	Simplified Adjusted			Patient Covariate Adjusted		
**Characteristic**	**OR**	**95% CI**	**p-value**	**OR**	**95% CI**	**p-value**
Date (Scaled)	0.63	0.58, 0.68	<0.001	0.72	0.64, 0.81	<0.001
Temperature (5 °C)	0.93	0.83, 1.04	0.2	0.92	0.78, 1.08	0.3
Precipitation (5 mm)	0.66	0.07, 6.02	0.7	2.74	0.14, 48.9	0.5
Temperature (5 °C) * Precipitation (5 mm)	1.06	0.72, 1.57	0.8	0.82	0.50, 1.39	0.5
Age (5 days)				1.00	1.00, 1.00	0.15
WHZ				0.87	0.81, 0.93	<0.001
HAZ				0.80	0.75, 0.85	<0.001
